# Effects of lightning on trees: A predictive model based on in situ electrical resistivity

**DOI:** 10.1002/ece3.3347

**Published:** 2017-09-12

**Authors:** Evan M. Gora, Phillip M. Bitzer, Jeffrey C. Burchfield, Stefan A. Schnitzer, Stephen P. Yanoviak

**Affiliations:** ^1^ Department of Biology University of Louisville Louisville KY USA; ^2^ Department of Atmospheric Science University of Alabama in Huntsville Huntsville AL USA; ^3^ Department of Biological Sciences Marquette University Milwaukee WI USA; ^4^ Smithsonian Tropical Research Institute Balboa Republic of Panama

**Keywords:** abiotic factors, disturbance, lianas, mortality, Panama

## Abstract

The effects of lightning on trees range from catastrophic death to the absence of observable damage. Such differences may be predictable among tree species, and more generally among plant life history strategies and growth forms. We used field‐collected electrical resistivity data in temperate and tropical forests to model how the distribution of power from a lightning discharge varies with tree size and identity, and with the presence of lianas. Estimated heating density (heat generated per volume of tree tissue) and maximum power (maximum rate of heating) from a standardized lightning discharge differed 300% among tree species. Tree size and morphology also were important; the heating density of a hypothetical 10 m tall *Alseis blackiana* was 49 times greater than for a 30 m tall conspecific, and 127 times greater than for a 30 m tall *Dipteryx panamensis*. Lianas may protect trees from lightning by conducting electric current; estimated heating and maximum power were reduced by 60% (±7.1%) for trees with one liana and by 87% (±4.0%) for trees with three lianas. This study provides the first quantitative mechanism describing how differences among trees can influence lightning–tree interactions, and how lianas can serve as natural lightning rods for trees.

## 1 INTRODUCTION

Lightning strikes thousands of trees each day (Taylor, 1974), and ca. 500 million hectares of forest exist in regions with high lightning frequency (i.e., >30 flashes km^−2^ year^−1^; (Christian et al., 2003; Albrecht, Goodman, Buechler, Blakeslee, & Christian, 2016)). The dramatic effects of lighting on trees have interested scientists for more than a century (Anonymous, 1898; Komarek, 1964; Stone, 1914; Taylor, 1977), but the spatial and temporal stochasticity of lightning remain major obstacles in the comprehensive understanding of its ecological significance (Knight, 1987; Mäkelä, Mäkelä, Haapalainen, & Porjo, 2016; Yanoviak et al., 2015). Clearly lightning often kills trees directly or indirectly (e.g., via fire or subsequent fungal and beetle infestations; (Sharples, 1933; Hodges & Pickard, 1971)). However, tree mortality rates remain unknown for most forests (Franklin, Shugart, & Harmon, 1987; Shugart, 1987; Stephenson et al., 2011), and the different mechanisms of individual tree death rarely are quantified. This is particularly problematic for trees in the relatively large “standing dead” category (Carey, Brown, Gillespie, & Lugo, 1994), many of which are due to lightning. Resolving these ambiguities is increasingly important as lightning frequency is expected to increase in a warmer world (Romps, Seeley, Vollaro, & Molinari, 2014; Williams, 2005). Here, we explore how variation in a key trait—electrical resistivity—can explain the varied effects of lightning on trees (hereafter, “lightning–tree interactions”).

Whereas lightning is a frequent cause of tree mortality in some regions (Brünig, 1964; Covert, 1924; Reynolds, 1940; Yanoviak et al., 2015), many trees struck by lightning suffer no apparent ill effects (Orville, 1968; Stone, 1914; Taylor, 1977). The most parsimonious hypotheses to explain this variation focus on differences in lightning intensity and physiological or anatomical differences among struck trees. In particular, the size, location, and species identity of trees are presumed to be key factors (Baker, 1973; Taylor, 1964; Yanoviak et al., 2015). The potential role of tree species‐level traits remains especially ambiguous, with historical references to “starchy” oak versus “oily” beech trees differing in their attractiveness or response to lightning (Covert, 1924). Despite these and many others suggested patterns, the majority of evidence concerning the probability that any given tree will be damaged by lightning remains anecdotal and post hoc, mainly for logistical reasons (Mäkelä, Karvinen, Porjo, Mäkelä, & Tuomi, 2009; Yanoviak et al., 2015).

Lightning damages trees mainly through heat energy—both the extreme quantity of heat and the high rate at which it is applied to tree tissues (hereafter referred to as heating and maximum power, respectively; (Uman, 2008; Courty, 2017)). These two properties are proportional to the total current and peak current, respectively, of a lightning discharge. High peak current (typically 15–30 kA) causes high maximum power, that is, hypothesized to generate steam explosions in the vascular cambium. Such localized explosions create the stereotypical lightning scars on tree trunks, and sometimes catastrophically shatter entire trees (Mäkelä et al., 2009; Plummer, 1912; Stone & Chapman, 1912; Taylor, 1964). Similarly, a prolonged lightning discharge (i.e., “continuing current” or CC lightning, typically 200 A for 115 ms (Bitzer, 2017)) causes sustained heating that presumably kills trees and ignites forest fires (Anderson, 1964; Fuquay, Taylor, Hawe, & Schmid, 1972; Kitagawa, Brook, & Workman, 1962). What humans commonly perceive as a single lightning flash is actually a very complex phenomenon having three main properties: (i) the number of return strokes (visible pulses of electric current), (ii) the duration of the current in each return stroke, and (iii) the peak current of each return stroke (Uman, 2001). These properties are highly variable among flashes, potentially contributing to stochastic variation in lightning–tree interactions.

Variation in electrical resistivity among trees is also expected to affect the amount of heating and maximum power experienced during a lightning discharge (Komarek, 1964; Stone & Chapman, 1912). The amount of heating and maximum power are directly proportional to the electrical resistance (*R*) of the struck tree (Uman, 2008), which varies among tree species and their general morphology (the three‐dimensional shape of a tree, see Equation** 1 below). Specifically, electrical resistivity differs consistently among species and increases with tree diameter in all cases (Gora & Yanoviak, 2015). Such differences may explain why lightning‐caused tree deaths appear to be twice as common for relatively resistant conifers as they are for more conductive hardwoods (Baker, 1973; Reynolds, 1940; Taylor, 1977). Differences in tree size also are potentially relevant in this context; biomass increases exponentially with diameter for healthy trees, thus larger trees may survive lightning by distributing a similar amount of heat across more biomass.

Although many plant traits vary predictably with latitude (e.g., freeze tolerance, deciduousness), structural differences in vascular tissue between growth forms (trees and climbing plants) generally are consistent between temperate and tropical regions (Angyalossy, Pace, & Lima, 2015; Christensen‐Dalsgaard, Fournier, Ennos, & Barfod, 2007). Specifically, climbing plants typically hold more water per unit of stem volume than do trees in both temperate and tropical regions. Relative water content (and other factors, like ion content) partly determines the electrical resistance of plant tissues (Bieker & Rust, 2010; Stamm, 1927; Stone, 1914) and likely explains the lower resistivity of vines versus trees in the temperate zone (Gora & Yanoviak, 2015). However, similar comparative data do not exist for tropical plants.

Other factors extrinsic to lightning flash characteristics and tree traits also likely influence the extent of damage that occurs during a lightning discharge. Although hard evidence is lacking, lightning damage to trees may be influenced by soil type (Covert, 1924; Plummer, 1912), elevation (Muzika, Guyette, Stambaugh, & Marschall, 2015), or swampy conditions (Anderson, 1964). Recent observations indicate that another factor—the presence of lianas (woody vines)—influences the effect of lightning on trees (Yanoviak, 2013). Specifically, the tendency for liana stems to be more conductive than tree branches of similar diameter (Gora & Yanoviak, 2015; Yanoviak, 2013) suggest that lianas function as natural lightning rods. This effect should be particularly important in tropical forests, where lightning frequency is high and ca. 40% of the forest canopy is carpeted by liana foliage (Christian et al., 2003; Putz, 1984; Schnitzer et al., 2012).

The principal objective of this study was to determine how variation in electrical resistivity within and among trees and lianas could influence lightning–tree interactions. The electrical properties of tropical plants are unknown, so we quantified the electrical resistivity of some common woody plants in central Panama. We hypothesized that lianas would have lower resistivity than trees, as observed in temperate regions (Gora & Yanoviak, 2015). Because resistivity is linked to moisture content, we further hypothesized that differences in electrical resistivity between and within growth forms (lianas vs. trees) would correspond to differences in their relative water content. We explored how resistivity as a plant trait should affect the heating and maximum power experienced by trees during three common types of lightning discharges. Specifically, we predicted that heating and maximum power decrease with increasing tree size (increased height and diameter), and differ among tree species due to differences in their general morphology and electrical resistivity. Finally, we estimated the potential for lianas to reduce heating and power within host trees by diverting electric current. Our overall goal was to model the directional effects of tree characteristics on heating and maximum power as a basis for predicting the varied ecological effects of lightning.

## 2 METHODS

Field work for this project was conducted in the Barro Colorado Nature Monument (BCNM) in Panama (9.15°N, 79.85°W). The BCNM is a seasonally moist lowland tropical forest administered by the Smithsonian Tropical Research Institute. Additional information about this forest is available elsewhere (Leigh, Rand, & Windsor, 1996).

### 2.1 Electrical resistivity measurements

We selected six common species of trees and seven common species of lianas to measure differences in resistivity between growth forms, and among species within growth forms (Table 1). We measured only tree and liana stems 1–10 cm in diameter for the growth form comparison because liana stems larger than this size range are uncommon for most species (Schnitzer et al., 2012). To reduce confounding phylogenetic effects, we chose species that minimized phylogenetic similarity within the growth forms and maximized similarity between trees and lianas. Specifically, three pairs of lianas and trees were in the same taxonomic families, whereas all species within each growth form were in different families (Table 1). We also performed a separate comparison of larger stems (10–77 cm) for a subgroup of three tree species (*Alseis blackiana*,* N* = 19; *Dipteryx panamensis*,* N* = 12; and *Jacaranda copaia*,* N* = 20).

**Table 1:** List of the focal plant species used in this study

	Species	Family	<3 cm	3–10 cm	>10 cm
Trees (*N* = 145)	*Dipteryx panamensis*	Fabaceae	8	7	12
	*Jacaranda copaia*	Bignoniaceae	8	8	20
	*Terminalia amazonia*	Combretaceae	7	8	–
	*Luehea seemannii*	Malvaceae	5	8	–
	*Miconia argentea*	Melastomataceae	8	8	–
	*Alseis blackiana*	Rubiacaea	6	8	19
Lianas (*N* = 103)	*Clitoria javitensis*	Fabaceae	7	8	–
	*Arrabidaea patellifera*	Bignoniaceae	6	9	–
	*Combretum decandrum*	Combretaceae	8	8	–
	*Connarus panamensis*	Connaraceae	7	7	–
	*Davilla nitida*	Dilleniaceae	6	9	–
	*Hippocratea volubilis*	Celastraceae	7	8	–
	*Coccoloba parimensis*	Polygonaceae	11	7	–

Stems were divided into three groups based on diameter. Values are sample sizes (*N*) for each diameter class. All data were independent, that is, different stems were used for each measurement.

The field methods for this project followed those of Gora and Yanoviak (2015). Briefly, we measured the electrical resistance of stems or branches of lianas and trees (saplings or larger trees, hereafter all are referred to as *stems*) using a megaohmmeter (DR‐6605; Ruby Electronics, Saratoga, CA, USA) secured to two electrodes (aluminum nails). The electrodes were separated by 30 cm and inserted on the same longitudinal axis of a liana or tree stem. We measured diameter of the stem at the midpoint between the two electrodes and recorded air temperature. We then calculated electrical resistivity using Equation 1,

(1) $$p=\frac{RA}{L}$$

where *R* is resistance (ohms, Ω), *p* is resistivity (Ωm), *A* is cross‐sectional area (m^2^), and *L* is length (m) of the measured section. To avoid potentially confounding environmental effects, all measurements were taken during dry conditions and at consistent temperatures during peak lightning season (i.e., wet season; June–October). To verify that minor variation in electrode depth was not an important source of error, we measured resistance with electrodes inserted 1.5, 2.5, and 3.5 cm into the vascular tissues of two or more of the individuals for each of the 11 tree species used in the model (>30 individuals in total). Resistance was consistent regardless of probe depth over this range.

We used one focal species from each growth form (the liana *Arrabidaea patellifera*,* N* = 15; and the tree *A. blackiana*,* N* = 15) to quantify how resistivity changes with stem moisture content. We measured electrical resistance as described above, except that the electrodes were separated by 20 cm. After recording resistance, we removed the 20 cm section of stem using a handsaw and sealed it in a preweighed plastic bag. We then weighed each fresh stem section, dried it to constant mass in an oven at 60°C, and recorded its dry weight. Dry mass was subtracted from wet mass to calculate moisture mass and percent moisture content.

### 2.2 Heating and maximum power modeling

The amount of heating and maximum power generated in tree tissues during a lightning strike fundamentally are determined by stem resistance. Using 533 in situ measurements of resistivity, we modeled how heating and maximum power during a lightning strike differ within and among tropical and temperate tree species given different initial conditions (i.e., different lightning flash characteristics). The model included three types of lightning discharges, 11 species of trees, one temperate liana, and one tropical liana. We assumed no irregularities in tree morphology and no variation within each of the three types of lightning. We also assumed that the resistivity of plant tissues does not change during a lightning discharge, that heat is evenly distributed among tree tissues and not dissipated away from the tree during a discharge (which typically occurs in <1 ms), and that lightning current does not flashover to nearby objects. Finally, electric current flows longitudinally through tree vascular tissues regardless of the source (e.g., lightning or an ohmmeter; Carter & Blanchard, 1978; Smith & Blanchard, 1984; Taylor, 1974, 1977). Thus, we assumed that resistance measured by an ohmmeter is relatively similar to that encountered by lightning current. These assumptions were consistent for all model iterations. If some of these assumptions are violated then the magnitude of heating or maximum power will change, but the directional effects (e.g., whether lianas decrease heating) of tree characteristics are unlikely to be affected.

We used Equation 2 to compare the resistive heating (hereafter referred to as “heating”) of different tree species in response to each type of lightning discharge. In this equation, heating is equal to the action integral multiplied by the resistance of a tree:

(2) $$H=\int I^{2}\left(t\right)R\;dt$$

where *H* is total resistive heating of a tree (joules, J), *I* is current (amperes, A), *t* is the duration of the current in the lightning return stroke (seconds), and *R* is the resistance (Ω) of the selected tree (Uman, 2008). The action integral (*I* ^2^ × *t*) is specific to each type of lightning, and resistance differs among tree species, sizes (as trunk volume), and tree morphologies (as change in diameter with height). Thus, this formula can be used to calculate the heating of any free‐standing tree given the values for these two terms. Because the thermal properties of tree tissues are unknown for most species, we did not estimate increases in temperature as a result of heating. Similarly, we calculated maximum power by multiplying the squared peak current by the resistance of the tree. Time was excluded from this calculation because peak current is an instantaneous value. Hereafter, the heat values calculated using Equation 2 are referred to as *heating* (J), and calculated maximum power is referred to as *maximum power* (J/s). To facilitate the comparison of heating for different sizes of trees, we normalized heating by tree volume to determine the *heating density* (J/cm^3^).

We combined Equation (2) and Ohm's Law to quantify the potential for lianas to function as natural lightning rods. Lianas were conspicuously damaged by electric current in >90% of the lightning strikes on BCI (Yanoviak, Gora, Burchfield, Bitzer, & Detto, 2017) in a separate study, demonstrating that electric current flows through both trees and their resident lianas during a strike. Consequently, we assume that the electrical potential (voltage) across all main stems in a liana‐tree complex is the same during a lightning discharge. However, the proportion of lightning current flowing through each stem in the complex will differ according to its resistance (obtained from field measurements). Given this relationship, we modeled the distribution of electric current between liana and tree stems during a lightning discharge as the ratio of tree resistance to liana resistance. We then used the methods described above to calculate heating and maximum power in the tree‐liana complex. To estimate the protective effects of multiple lianas in a single tree, we substituted liana resistance in the above ratio with the combined resistance of all lianas as if connected in a parallel circuit.

### 2.3 Resistance calculations

Using the same 533 resistance measurements mentioned above, we constructed hypothetical trees and lianas similar to a model tree used in a previous lightning‐focused study (Defandorf, 1955). We approximated tree and liana shape as a conical stack of 1‐cm tall cylinders incrementally decreasing in diameter from the base to the top. This approach simulated the relatively linear path that electric current follows from the end of any given canopy branch to the base of the tree (Taylor, 1974). The top (minimum) diameter was fixed at 1 cm in all cases, and the incremental increase was calculated as (maximum diameter − minimum diameter)/height in centimeters. We determined the resistivity of each cylinder in the stack based on species‐specific logarithmic functions of resistivity versus diameter calculated from field data (Table 2; [also see Gora & Yanoviak, 2015]). Consequently, we estimated the heating and maximum power experienced by an average tree of each species. The resistivity of each cylinder was multiplied by height (i.e., 1 cm) and divided by its cross‐sectional area to determine resistance. This conversion makes no assumption about the composition of tissues within each cylinder, but rather assumes that electric current from the in situ resistance measurements follow a similar path in the model tree. We calculated total resistance and total volume as the summed resistance (as if in a series circuit) and volume, respectively, of all cylinders in a given tree or liana. We used total tree volume to estimate heating density (rather than estimating the volume and resistivity of specific tissues; (Al Hagrey, 2006)) because we assumed that heat is distributed evenly among tree tissues.

**Table 2:** Resistance, maximum power, and heating for 11 different tropical and temperate tree species, and three types of lightning flashes (D1, D2, D3 = Discharges 1, 2, and 3 as described in the text)

Region	Species	Resistivity‐diameter function		Resistance (kΩ)	Maximum power (TW)	Heating (GJ)		
		Slope	Intercept			D1	D2	D3
Tropical	*Jacaranda copaia*	8.29	2.07	1,275	1,154	18.9	27.6	24.7
	*Alseis blackiana*	8.66	2.39	2,062	1,867	30.6	44.7	40.0
	*Dipteryx panamensis*	8.77	1.87	1,284	1,163	19.0	27.9	25.0
Temperate	*Acer rubrum*	8.46	2.29	1,716	1,554	25.4	37.2	33.3
	*Acer saccharum*	8.17	2.83	2,615	2,368	38.8	56.7	50.7
	*Quercus rubra*	8.42	2.63	2,359	2,136	35.0	51.2	45.8
	*Betula alleghaniensis*	8.23	2.75	2,471	2,238	36.6	53.6	48.0
	*Pinus virginiana*	9.09	2.51	2,706	2,450	40.1	58.7	52.5
	*Pinus resinosa*	7.91	3.28	3,685	3,336	54.6	80.0	71.5
	*Pinus strobus*	8.66	2.55	2,406	2,178	35.7	52.2	46.7
	*Tsuga canadensis*	8.89	2.41	2,306	2,088	34.2	50.0	44.8

Maximum power is the same for all three types of lightning. All model trees were 20 m tall with a minimum diameter of 1 cm at their top and a basal diameter of 27.3 cm. Resistivity for each tree was calculated using the resistivity‐diameter function: ln (*p*) = *mD* + b, where *p* and *D* are resistivity and the cube‐root of diameter, respectively.

We compared heating and maximum power among 11 tree species—the three tropical tree species in this study and eight temperate species surveyed in a separate study (Table 2; Gora & Yanoviak, 2015). We used 20 m as tree height for interspecific comparisons because mature canopy trees in temperate and tropical forests tend to be at least that tall (Mascaro et al., 2011). We calculated maximum diameter at ground level using height–to–diameter ratios of *Prioria copaia*, which is the only common emergent tropical tree in the BCNM for which such data exist (O'Brien, Hubbell, Spiro, Condit, & Foster, 1995).

We used region‐specific liana data to estimate the effectiveness of lianas as natural lightning rods. Specifically, we created hypothetical tropical and temperate lianas using resistivity data from liana stems measured in Panama and Kentucky (Gora & Yanoviak, 2015), respectively. We conservatively used 6 cm as the maximum liana diameter for the model. Many lianas with greater diameter reside in the canopy on BCI (Kurzel, Schnitzer, & Carson, 2006); thus, the size of our model liana underestimates their potential protective effects. We also assumed that liana stems are 25% longer than their host tree height due to their sinuous growth and scandent habit. We calculated the reduction in heating and maximum power for trees supporting either one or three lianas. We chose this range because it reflects actual liana abundances in trees of the BCNM (Putz, 1984).

Intraspecific tree size comparisons focused on *D. panamensis* and *A. blackiana*. We chose *D. panamensis* because it is the closest relative of *P. copaia* in the suite of focal species used for this study, and because both species have similar general morphology. We chose *A. blackiana* because its morphology is distinct from *D. panamensis*. Height–to–diameter ratios were determined using the same methods for both species (O'Brien et al., 1995), and we used these parameters to calculate the heating and maximum power for seven sizes of each species (10, 15, 18, 20, 22, 25, and 30 m).

### 2.4 Lightning current profiles

It is impractical to model every possible type of lightning, so we focused on three common canonical lightning discharges (single stroke, multiple stroke, and continuing current) to capture a range of the potential energetic effects of lightning on trees. In each hypothetical discharge, the current at the strike point (i.e., the tree) was estimated using a binomial exponential model (Diendorfer & Uman, 1990; Heidler, 1985; Heidler & Cvetic, 2002) in which each term is of the form:

$$i\left(t\right)=\frac{I_{0}}{\mu }*\frac{{\left(\frac{t}{\tau_{1}}\right)}^{2}}{{\left(\frac{t}{\tau_{1}}\right)}^{2}+1}*\exp\left(-\frac{t}{\tau_{2}}\right)$$

where *I*_0_ is the current amplitude, μ is an amplitude correction factor, and $\tau_{1},\tau_{2}$ are decay time constants.

We created three different types of hypothetical lightning discharges based on Diendorfer and Uman (1990). The simplest type, *Discharge 1*, was a single cloud‐to‐ground (CG) return stroke discharge with a peak current of 30 kA. These, and other parameters, are the same as the CURRENT‐2 flash in Table 1 of Diendorfer and Uman (1990). Because most CG lightning discharges contain more than one stroke, we created hypothetical *Discharge 2* with three return strokes. In this case, the first return stroke was the same as *Discharge 1*, but the second and third strokes followed the parameters of the CURRENT‐1 flash in Table 1 of Diendorfer and Uman (1990). Finally, we modeled *Discharge 3* as a CC flash (Bitzer, 2017; Kitagawa et al., 1962) that included a single stroke (the same as *Discharge 1*) of 50 μs duration immediately followed by a constant current of 200 A for 115 ms.

### 2.5 Statistical analyzes

We used analysis of covariance (ANCOVA) to test for differences in resistivity among growth forms and species. Preliminary examination of the data revealed conspicuous heteroscedasticity. Specifically, variance in resistivity was much greater for stems <3 cm diameter than for larger stems. Consequently, we ran separate analyses for stems <3 cm and stems 3–10 cm. We tested for differences in resistivity between growth forms using species nested within growth form. When resistivity differed between growth forms, we tested for interspecific differences in resistivity within each growth form separately. Stem diameter was the covariate in all of these tests.

We ran a series of comparable analyzes to test the hypothesis that differences in resistivity among stems are associated with variation in their relative moisture content. We used ANCOVA to determine how resistivity differed between species using stem diameter as the covariate. We repeated this analysis using moisture content as the covariate, and we used regression to determine how resistivity changed with moisture content independent of species. Finally, we compared the squared residual error of linear models with and without species as a fixed effect to determine whether the relationship between resistivity and moisture content was species‐independent.

In all cases, we used stepwise model reduction to remove nonsignificant interaction terms and we present statistical results from these reduced models. We did not include temperature in our analyses because it was relatively consistent (see Section 3), and differences in temperature much larger than those observed here were unimportant in a similar study (Gora & Yanoviak, 2015). We used the Bonferroni correction for multiplicity when necessary. Electrical resistivity data were log‐transformed, and diameter was cube‐root transformed to improve linear relationships among these variables. We used the Shapiro–Wilk test to assess normality, and we examined residuals to confirm appropriate model fit.

Data were analyzed using the R statistical program (R Development Core Team, 2016). We used the *lme4* package with the *LmerTest* modification to analyze mixed‐effect models (Bates, Maechler, Bolker, & Walker, 2014) and the base R package for basic linear models and *t* tests. We tested for differences among individual species using post hoc Tukey HSD test in the *multcomp* package (Hothorn, Bretz, & Westfall, 2008).

## 3 RESULTS

**Figure 1:**
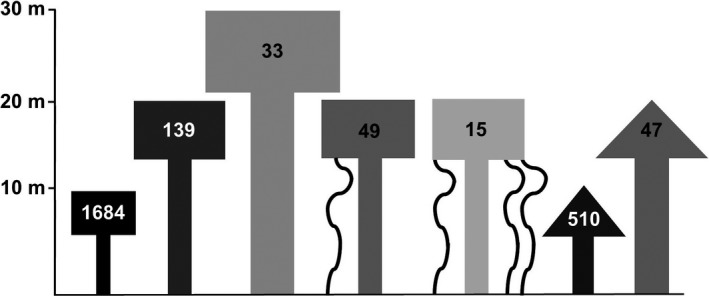
The canopy profile of a hypothetical tropical forest composed of *Dipteryx panamensis* (triangular tree crowns) and *Alseis blackiana* (rectangular tree crowns). The gray shade of each model tree and the superimposed number indicate heating density (kJ/cm^3^). Gray shades span a gradient from “hot” (dark gray) to “cool” (light gray), indicating high and low levels of heating during a lightning discharge, respectively. Lianas are represented as sinuous structures descending from two of the trees. Tree height and relative trunk diameters are drawn to scale. Heating density is affected by tree species, tree height, tree diameter, and the presence of lianas (see text for details)

The model supported the prediction that variation in resistivity among basic tree characteristics is likely to influence lightning–tree interactions. Specifically, the amount of heating and maximum power (i.e., the amount of tissue damage) expected to occur during a lightning discharge differed among tree species, sizes, and tree morphologies, and with the abundance of lianas (Figure 1). The model predicted that hypothetical trees experience heating from 3 to 80 GJ, heating density from 8 to 1,685 kJ/cm^3^, and maximum power of 197–3,336 TW. For clarity, hereafter we focus on tree interactions with a single, non‐CC return stroke (*Discharge 1*).

**Figure 2:**
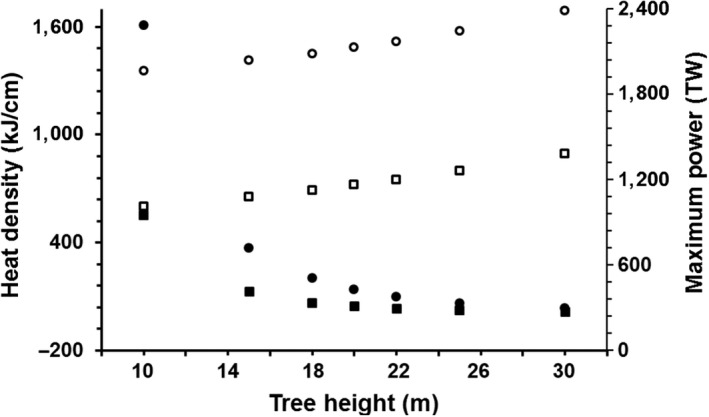
Predicted changes in heating density (filled shapes) and maximum power (unfilled shapes) during a lightning discharge versus the height of hypothetical *Dipteryx panamensis* (squares) and *Alseis blackiana* (circles) trees

Predicted lightning–tree interactions differed among species and tended to be more severe for temperate trees. Interspecific differences in heating and maximum power were caused by variation in both the resistivity of stem tissues and overall morphology. When considering only resistivity, heating was lowest for the tropical trees *J. copaia* (18.9 GJ) and *D. panamensis* (19.0 GJ), whereas heating of *A. blackiana* (30.6 GJ) was ca. 60% greater than for either of these species (Table 2). Temperate trees typically had greater estimated heating than tropical trees. Specifically, heating of the tropical tree *A. blackiana* was lower than all temperate species except for *Acer rubrum* (25.4 GJ), and heating of the remaining seven temperate species was 81%–189% greater than *J. copaia* (Table 2). After accounting for variation in trunk morphology as well (*A. blackiana* is narrower), heating density and maximum power of *A. blackiana* were 290% and 180% greater than for *D. panamensis*, respectively (Tables 2 and 3, Figure 2). Tree morphology was a species‐specific property in this study, but differences in the shape of branches within the same species or even the same individual should similarly affect patterns of heating and maximum power.

**Table 3:** Total heating, both as an absolute value and per volume of tissue, and maximum power among different sizes of hypothetical *Dipteryx panamensis* and *Alseis blackiana* trees

Species	Height (m)	Maximum diameter (cm)	Resistance (kΩ)	Volume (m^3^)	Maximum Power (TW)	Total heating (GJ)	Heat density (kJ/cm^3^)
*Dipteryx panamensis*	30	47.4	1,525	1.80	1,380	22.6	12.5
	25	37.0	1,394	0.92	1,262	20.7	22.5
	22	31.1	1,326	0.58	1,200	19.6	34.2
	20	27.3	1,284	0.40	1,163	19.0	47.0
	18	23.7	1,244	0.28	1,126	18.4	66.7
	15	18.5	1,190	0.14	1,077	17.6	124.3
	10	10.6	1,114	0.03	1,009	16.5	509.7
*Alseis blackiana*	30	38.3	2,633	1.18	2,384	39.0	33.0
	25	29.5	2,477	0.59	2,243	36.7	62.3
	22	24.6	2,396	0.36	2,169	35.5	97.8
	20	21.4	2,352	0.25	2,130	34.9	138.7
	18	18.5	2,302	0.17	2,085	34.1	200.4
	15	14.2	2,251	0.09	2,038	33.4	392.1
	10	8.0	2,171	0.02	1,966	32.2	1684.8

The minimum diameter at the top of each tree was defined as 1.0 cm, and maximum diameter was determined using different height:diameter relationships for each species as explained in the text.

Within a species, taller model trees experienced greater heating and maximum power, yet their heating density was substantially lower (Figure 2, Table 3). For *A. blackiana* trees, the maximum power expected for a 30 m tall individual (2,384 TW) was 21% greater than for a 10 m individual (1,966 TW). Taller trees also experience more total heating, but the heat is distributed over a larger volume of tree tissue, effectively reducing the impact of lightning. For example, heating density for a 10 m tall individual of *A. blackiana* (1,684 kJ/cm^3^) was ca. 49 times greater than for a 30 m tall individual (33 kJ/cm^3^). These size‐based differences compounded the interspecific resistivity‐based differences described above. Specifically, the heating density of a 10 m tall *A. blackiana* tree was ca. 127 times greater than the heating density of a 30 m tall *D. panamensis* (Figure 2).

**Figure 3:**
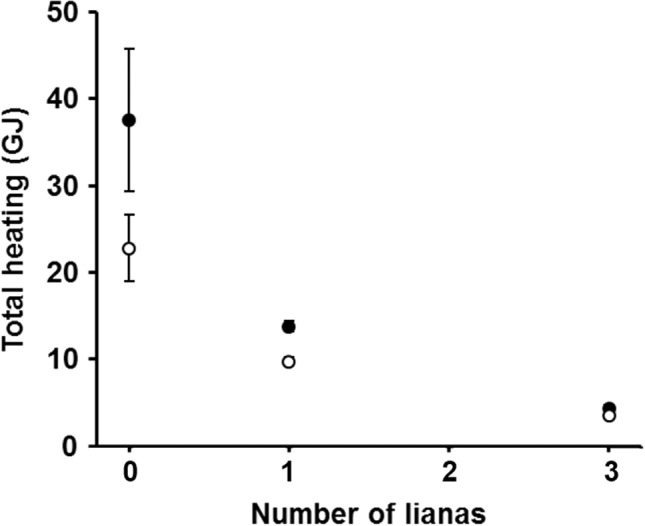
Predicted total heating (mean ± *SE*) of temperate (filled circles, *n* = 8 species) and tropical (unfilled circles, *n* = 3 species) trees versus the number of lianas present in each. Predicted maximum power follows the same pattern

Inclusion of lianas in the model dramatically reduced the heating and maximum power experienced by their host trees, suggesting that lianas have the capacity to inadvertently protect trees from lethal lightning damage (Figure 3, Table 4). The presence of one liana reduced both heating and maximum power by more than half (Figure 3; mean ± *SD*: 60.4 ± 7.1% reduction). This protective effect increased when more lianas were added; three lianas on a single tree reduced heating and maximum power by 87% (±4.0%). The expected protective effect of lianas was higher in trees with greater electrical resistance (e.g., larger individuals or relatively resistant species) because the lianas diverted a larger fraction of the total lightning current. For example, as described above, a liana‐free *A. blackiana* tree should be more heavily damaged by lightning than other liana‐free tropical trees. However, adding three lianas to an *A. blackiana* would cause it to have the lowest heating and maximum power among all of the modeled species. Similarly, more conductive lianas, such as those with larger diameters, would divert more lightning current and thus provide greater protection for host trees.

**Table 4:** The predicted decrease in heating and maximum power experienced by trees with 0, 1, or 3 lianas present (0L, 1L, and 3L)

Region	Species	Total heating (GJ)			Maximum power (TW)			Total heating or power diverted (%)	
		0L	1L	3L	0L	1L	3L	1L	3L
Tropical	*Jacaranda copaia*	18.9	9.27	3.63	1,154	566	222	51	81
	*Alseis blackiana*	30.6	10.7	3.23	1,867	653	197	65	89
	*Dipteryx panamensis*	19	9.3	3.62	1,163	569	222	51	81
Temperate	*Acer rubrum*	25.4	12.6	4.79	1,554	771	293	50	81
	*Acer saccharum*	38.8	14	4.34	2,368	854	265	64	89
	*Quercus rubra*	35	13.7	4.48	2,136	836	273	61	87
	*Betula alleghaniensis*	36.6	13.9	4.42	2,238	850	270	62	88
	*Pinus virginiana*	40.1	14.1	4.29	2,450	862	262	65	89
	*Pinus resinosa*	54.6	14.6	3.78	3,336	892	231	73	93
	*Pinus strobus*	35.7	13.8	4.46	2,178	842	272	61	88
	*Tsuga canadensis*	34.2	13.6	4.51	2,088	830	275	60	87

Values are based on a single‐stroke lightning flash (*Discharge 1* in the text). Lianas divert an equal proportion heat and power away from the tree stem, thus the percentages are only presented once.

Finally, variation in discharge types strongly affected the predicted heating experienced by the model trees. Relative to the single stroke event (*Discharge 1*), heating was 45% higher for the three stroke flash (*Discharge 2*), and ca. 31% higher for the continuing current flash (*Discharge 3*; Table 2). By contrast, maximum power was equal for all three types of lightning because each had the same peak current. Heating and maximum power are proportional to tree resistance; thus, relative differences among species were the same for any type of lightning discharge.

### 3.1 Electrical resistivity of tropical plants

**Figure 4:**
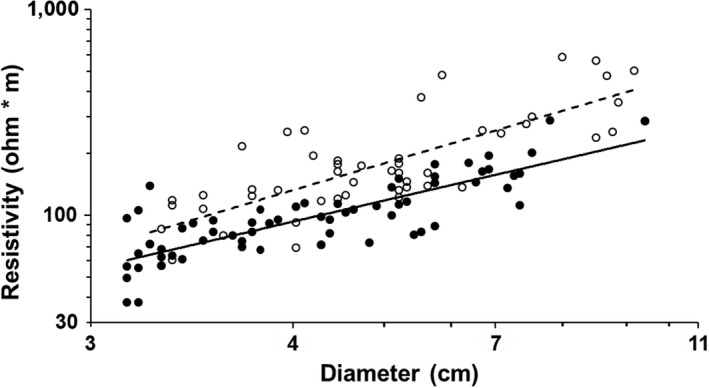
Electrical resistivity versus diameter for tree (open circles, dashed line) and liana (solid circles, solid line) stems 3–10 cm in diameter. Note that the *x*‐axis is cube‐root transformed

**Figure 5:**
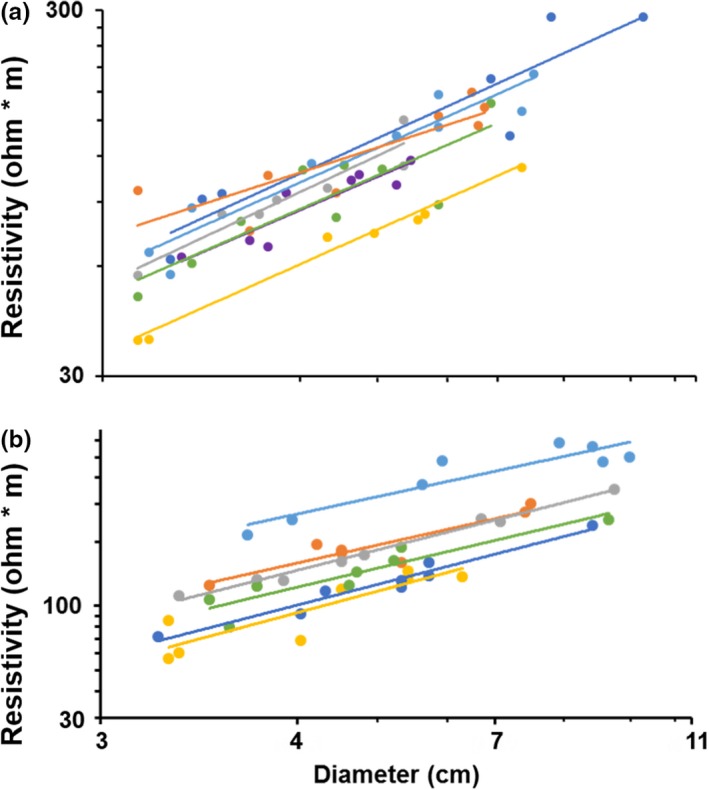
Electrical resistivity of stems 3–10 cm in diameter for various liana (a) and tree (b) species. The *x*‐axis is cube‐root transformed. Note that the *y*‐axis scales differ between the two plots. Within b, the different colored data points and regression lines refer to different species from top to bottom as follows: *Alseis blackiana* (light blue), *Dipteryx panamensis* (orange), *Jacaranda copaia* (gray), *Terminalia amazonia* (green), *Miconia argentea* (dark blue), and *Luehea seemannii* (yellow). Within plot a, the different points and regression lines refer to liana species from top to bottom as follows: *Connarus panamensis* (dark blue), *Arrabidaea patellifera* (light blue), *Hippocratea volubilis* (orange), *Coccoloba parimensis* (gray), *Davilla nitida* (green), *Clitoria javitensis* (purple), and *Combretum decandrum* (yellow)

Electrical resistivity generally differed between lianas and trees; liana resistivity was on average ca. 50% lower than that of trees for stems 3–10 cm in diameter (*F*_1,103_ = 7.01, *p* = .023, α = .025; Figure 4). By contrast, electrical resistivity did not differ between liana and tree stems <3 cm diameter (*F*_1,94_ = 0.937, *p* = .336). Temperature at the time of measurement was similar between growth forms (mean ± *SD* = 28.4 ± 1.8°C; *F*_1,240_ = 2.98, *p* = .08), and electrical resistivity increased with diameter in all cases (Figures 4 and 5).

**Figure 6:**
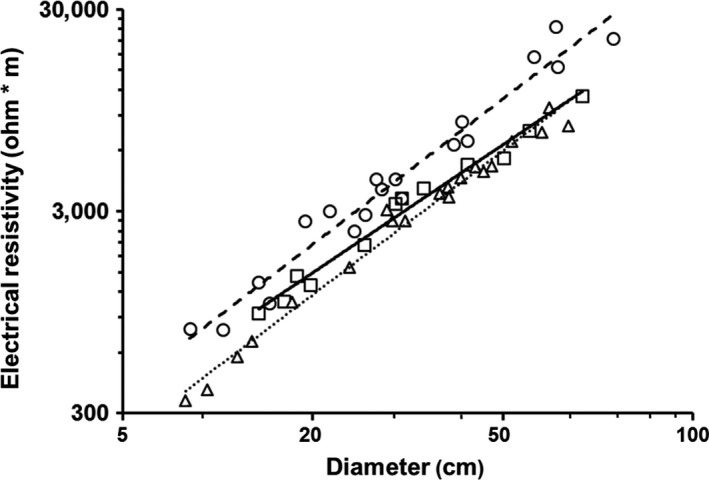
Electrical resistivity versus diameter for tree stems >10 cm in diameter (*Alseis blackiana* = circles, dashed line; *Dipteryx panamensis* = squares, solid line; and *Jacaranda copaia* = triangles, dotted line). The *x*‐axis is cube‐root transformed

As with temperate plants, electrical resistivity differed interspecifically within tropical trees and lianas for stems 3–10 cm in diameter (trees: *F*_5,40_ = 115.16, *p* < .001; lianas: *F*_6,48_ = 22.03, *p* < .001; α = .025; Figure 5). Electrical resistivity also differed among tree species for stem diameters >10 cm (*F*_3,47_ = 567.2, *p* < .001; Figure 6). Regardless of stem size, *A. blackiana* had the highest resistivity by a substantial margin, whereas the resistivity of *D. panamensis* was either similar to (stems 3–10 cm) or slightly higher (stems > 10 cm) than that of *J*. *copaia*. We lacked sufficient data for similar post hoc tests among liana species.

**Figure 7:**
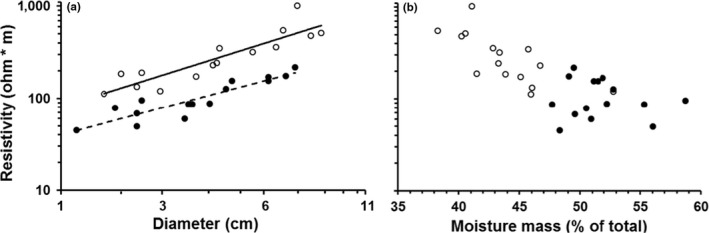
Resistivity across a range of diameter (a) and moisture content (b) for the same individuals of *Alseis blackiana* (solid line and open circles) and *Arrabidaea patellifera* (dashed line and filled circles). The *x*‐axis is cube‐root transformed in panel a

Differences in moisture content likely are driving the differences in resistivity described above. Electrical resistivity of *Arrabidaea patellifera* and *A. blackiana* increased with diameter (*F*_2,27_ = 112.2, *p* < .001, Figure 7a), but decreased with increasing moisture content (*F*_2,27_ = 19.0, *p* < .001, Figure 7b). *Alseis blackiana* consistently had higher resistivity than *A. patellifera* across a range of diameters (*F*_2,27_ = 116.0, *p* < .001), but their ranges of moisture content largely did not overlap (Figure 7). Variation in electrical resistivity was minimal above 55% moisture content, indicating that extremely wet stems exhibit a different relationship between resistivity and moisture content. Regardless, the strongest evidence that patterns of resistivity are driven by moisture content is that moisture was a species‐independent predictor of resistivity. That is, when the species term was dropped from the linear model for moisture content versus resistivity, the *R* ^2^ remained 0.55 (moisture with species: *F*_2,27_ = 19.00, *p* < .001; moisture without species: *F*_1,28_ = 35.8, *p* < .001, Figure 7b).

## 4 DISCUSSION

There is a long history of speculation regarding the differential effects of lightning among trees based on size, species, condition, and location (Anderson, 1964; Anonymous, 1898; Covert, 1924). Here, we present the first quantitative, mechanistic, predictive foundation for understanding how any healthy tree potentially will be affected by lightning. Unlike all previous work on this topic, the modeled effects of lightning on trees in this study are based on empirical measurements of an emergent physical property (electrical resistivity), which varies consistently with tree species and morphology. Although every strike event is unique, and its consequences ultimately are influenced by many factors that are not easily quantified, this model provides a straightforward and ecologically relevant starting point. Most importantly, it shows how differences in basic characteristics of trees can cause substantial differences in the amount of damage they experience from a lightning strike, *ceteris paribus*. Note that the model does not account for factors affecting the probability that any tree will be struck. For example, the effects of tree height illustrated in Figure 1 could be less important in mature forests if large trees are more likely to intercept lightning strikes. However, the model does suggest that trees in regenerating secondary forests will have relatively higher rates of severe or lethal lightning‐caused damage by virtue of their smaller average size.

The results of this study provide correlative support for the hypothesis that lianas function as passive lightning protection for trees (Yanoviak, 2013). Lianas generally are considered to be structural parasites of trees (Stevens, 1987); thus, this potential protective role adds a new perspective on liana–tree interactions. Some tropical trees often are liana‐free by the time they grow to canopy or emergent height (pers. obs.), and the results of this study suggest that lightning could contribute to that pattern by killing lianas in large, relatively conductive trees. Ultimately, uncovering such patterns will require experimental manipulation of lightning strike locations in a forest, or on accurate determination of lightning attachment locations across large areas of the forest canopy.

The tendency for lianas to have lower resistivity than trees likely reflects differences in moisture content between growth forms. Like Stamm (1927), we found that wood moisture content can supersede species identity as a determinant of electrical resistivity. Although the important role of moisture in wood resistivity is well established (Al Hagrey, 2006; Carter & Blanchard, 1978; Gora & Yanoviak, 2015), no other studies have compared moisture‐resistivity patterns among growth forms or trees in situ.

The model developed in this study also indicates that small trees will suffer more damage from a lightning strike than nearby larger trees. This pattern is supported by our observations of more than a dozen recent strikes in the forest on BCI, but post hoc assessments of lightning damage in other forests provide mixed evidence for differential mortality among tree size classes (Anderson, 1964; Magnusson, Lima, & de Lima, 1996). These latter studies were conducted months or years after the strike; thus, counts of dead stems could be biased against smaller size classes due to their lower persistence (Magnusson et al., 1996). Regardless, accurate field data collected within a few weeks after a strike are required to adequately test the relevance of tree size and other characteristics to the distribution of damage.

The estimates generated in this study show that multistroke and CC flashes produce more heat than the hypothetical single‐stroke flash (*Discharge 1*) used to generate the bulk of the heating and power estimates. In reality, ca. 80% of flashes have multiple (typically 3–5) return strokes, and ca. 40% are CC flashes (Bitzer, 2017; Rakov & Uman, 2003). Moreover, maximum peak current can be as much as 10 times greater than our model lightning discharges (300 kA instead of 30 kA). CC flashes can last up to 1 s (the modeled CC flash was 115 ms), and some discharges include >25 strokes (Uman, 2001). Consequently, extrapolating the model to large spatial or temporal scales likely would underestimate the damage.

Finally, the results of this study are potentially relevant to understanding future forest dynamics. Specifically, the model indicates that the likelihood of lightning‐caused death will be higher for tree species with high resistivity, smaller overall size, and relatively narrow trunks and branches. The relevance of these patterns depends on the probability that any given tree will be struck by lightning, and the relative importance of lightning as an agent of tree mortality at the population and community levels, which remains undetermined for most forests. However, given the high frequency of lightning in the lowland wet tropics, we suspect that its contribution to canopy tree mortality in particular is underestimated. Regardless, resolving this problem is important because lightning frequency is expected to increase over the coming decades (Romps et al., 2014; Williams, 2005).

This study raises at least four potentially fruitful avenues for future research. First, the simple conical shapes of the model trees and lianas ignored the diverse and often species‐specific three‐dimensional architecture of their natural counterparts. However, the model could be modified in future studies to more realistically account for differences in crown shape and complexity. Second, the model predictions and assumptions could be tested with high voltage experimental discharges in the laboratory (Wakasa, Nishimura, Shimizu, & Matsukura, 2012). Such tests could also determine the effects of nonuniform distribution of current (and subsequent damage) on tree survival or the production of lightning scars, and would provide insight into the damaging effects of extreme heating and power on living plant tissues under a variety of conditions. Third, fully testing the model will require large amounts of data on the real‐time distribution of CG lightning flashes, their characteristics, and their effects on trees, lianas, and other forest canopy elements. Such data are very difficult to obtain due to limitations in the spatial accuracy of lightning detection networks (Mäkelä et al., 2016), but advances in lightning sensing technology (Bitzer et al., 2013) suggest that this logistical hurdle soon will be overcome (Yanoviak et al., 2017). Finally, an accurate estimate of lightning‐caused death also fundamentally depends on the probability that any given tree will be struck by lightning. Incorporating this risk‐based information into the model would enhance its predictive power and broaden its applicability.

## CONFLICT OF INTEREST

None declared.

## AUTHOR CONTRIBUTIONS

EG designed and performed data collection, analyzed the data, helped design the model, drafted the manuscript, and coordinated the study; PB conceived the study, designed the model, and helped draft the manuscript; JB helped design the model and calculated model parameters; SS helped design data collection and edited the manuscript; SY initiated the collaboration, interpreted the results, and helped draft the manuscript. All authors gave final approval for publication.
